# Morphine in Combination with Ketamine Improves Cervical Cancer Pain and Suppresses Immune Function via the JAK3/STAT5 Pathway

**DOI:** 10.1155/2022/9364365

**Published:** 2022-04-21

**Authors:** Yurong Jiang, Tong Li, Yi Qian, Xiaoming Zuo, Jinmei Liu

**Affiliations:** ^1^Department of Anesthesiology, Jianhu People's Hospital of Jiangsu Province, Yancheng City, Jiangsu Province, 224700, China; ^2^Department of Gynecology, Jianhu People's Hospital of Jiangsu Province, Yancheng City, Jiangsu Province, 224700, China

## Abstract

**Background:**

The role of ketamine as an adjuvant for morphine in the treatment of cancer pain and immune functions has been confirmed. This study aimed to explore the role of morphine and ketamine on cancer pain and T cells of patients with cervical cancer (CC).

**Methods:**

T cells were isolated from peripheral blood mononuclear cells (PBMC) of CC patients by positive selection using anti-CD3 beads. The isolated T cells were assigned into three groups: the control group, the morphine group, and the morphine + ketamine (Mor + Ket) group. The percentages of CD4^+^ and CD8^+^ were analyzed by flow cytometry. The levels of interferon (IFN)-*γ*, interleukin (IL)-2, and IL-17 and the corresponding mRNA expression in vitro were determined using ELISA and qRT-PCR, respectively. Western blotting was used for detection of JAK3/STAT5 pathway-related proteins after naltrexone treatment in vitro. Afterwards, all the patients were further divided into the morphine group and the Mor + Ket group in accordance with the principles of the randomized and double-blind method to assess pain intensity.

**Results:**

Our in vivo results showed that drug combinations relieved cancer pain more effectively than morphine intervention. The in vitro results demonstrated that the combination of morphine and ketamine may decrease CD4^+^ percentage, CD4^+^/CD8^+^ ratio, and the levels of IFN-*γ*, IL-2, and IL-17 via the JAK3/STAT5 pathway.

**Conclusions:**

Our finding indicated that morphine-ketamine combination could improve cancer pain and repress immune function via the JAK3/STAT5 pathway in the progression of CC.

## 1. Introduction

Cervical cancer (CC) is a malignant gynecological cancer with relatively high morbidity and mortality in female representative tumors [1]. It is statistical that there are approximately 570000 patients newly diagnosed as CC worldwide in 2018, which accounts for 4% of all cancer patients [2]. The five-year survival rate of CC patients is only 17% [3] due to the rapid metastasis, immune dysfunction, and other cancer complications [4–6].

Generally, the course of cancer is accompanied by chronic pain, which is a serious problem for cancer patients and remarkably affects their quality of life (QOL) [7]. Therefore, except for the therapies such as surgery, chemotherapy, and radiotherapy to control the proliferation and metastasis of cancer [8, 9], pain relief may be beneficial for improving the patient's QOL while also supplementing cancer therapy. Nowadays, the inhibitory effect of opioids such as morphine on cancer pain has attracted much attention in clinical application [10–12]. For instance, Zheng et al. believed that long-term and low-dose of morphine can effectively attenuate moderate cancer pain [10]. Similarly, Barton reported that first-line low-dose morphine is better for the control of moderate cancer pain than weaker opioids [11]. Matsuoka et al. performed a prospective study on morphine and demonstrated that patients with cancer pain receive morphine titration and the pain intensity is remarkably alleviated on the 8^th^ day [12]. However, some adverse effects such as the dysfunction of immune function are also uncovered in the progression of morphine application. As an immunosuppressor, morphine can suppress CD4^+^ percentage and CD4^+^/CD8^+^ ratio [13–15], decrease the activity of natural killer cells [13–15], and inhibit the secretion of interferon (IFN)-*γ* and interleukin (IL)-2 [16–18]. For patients with cancer pain, these immunosuppressive effects may enhance the difficulty of cancer therapy.

Ketamine, a kind of nonopioid analgesic, generally acts as an adjuvant to opioids for cancer pain [19–21]. Zhou et al. combined morphine with ketamine in the treatments of refractory cancer pain and found that drug combinations effectively decrease the levels of IL-2 and IFN-*γ* in T cell isolated from patients with cancer pain [19]. In addition, morphine in combination with ketamine decreases the immune functions of patients with refractory cancer pain, and this inhibitory effect has no significant difference with morphine alone [20]. Hou et al. conducted an in vitro experiment in gastric cancer patients and conferred that addition of ketamine may be helpful for the reduction of morphine consumption and the relief of immunosuppression [21]. However, research on the accurate role of morphine combined with ketamine for the treatments of CC pain and the possible action mechanism is relatively rare.

In this study, the therapeutic effect of morphine combined with ketamine on cancer pain for CC patients and the relationships with the JAK3/STAT5 pathway on the regulation of immune functions were investigated. Our results uncovered a downstream action pathway of morphine combined ketamine on immune regulation and provided some theoretical bases for CC therapies in clinical practice.

## 2. Methods

### 2.1. Patients

This study included 20 patients with CC (age range: 45–65 years old; body mass index range: 18–25 kg/m^2^). The exclusion criteria were drugs abuse, diabetes complications, history of systemic inflammatory diseases and immunodeficiency diseases, and patients received treatments such as immunosuppression and chemoradiotherapy before admission. All patients were diagnosed as CC by histological examinations and underwent routine blood tests. The present study was approved by Jianhu People's Hospital's Ethics Committee (code: JY-LL-202004-J039) and strictly performed in accordance Declaration of Helsinki. The relevant informed consents were signed by each patient.

### 2.2. T Cell Isolation, Culture, and Grouping

The T cells were isolated from peripheral blood mononuclear cells (PBMC) by positive selection using anti-CD3 beads (Miltenyi Biotec, Inc.) and further confirmed by fluorescence-associated cell sorting (85% purity). The isolated T cells were cultured in RPMI 1640 containing 10% FBS and 1% streptomycin/penicillin at 37°C with 5% CO_2_.

The isolated T cells were assigned into three groups: the control group (normal saline), the morphine group (200 ng/mL), and the morphine + ketamine (Mor + Ket) group (200 ng/mL morphine + 100 ng/mL ketamine).

### 2.3. Flow Cytometry Analysis

After treatment for 24 h at 37°C, FITC-conjugated anti-CD3 (eBioscience, Inc, San Diego, CA, USA), APC-conjugated anti-CD4 (eBioscience), and PE-conjugated anti-CD8 (eBioscience) were incubated with the cells of each group for 1 h at 4°C. Afterwards, the cells were fixed with 1% paraformaldehyde and washed. The percentages of CD3^+^CD4^+^ and CD3^+^CD8^+^ were calculated using a flow cytometer (BD Biosciences, Franklin lakes, NJ, USA).

### 2.4. Measurement for the Levels of IL-2, IFN-*γ*, and IL-17

The levels of IL-2, IFN-*γ*, and IL-17 were measured by the corresponding commercial ELISA kits (R&D Systems, Minneapolis, MN, USA). Meanwhile, the relative expression of these cytokines was further detected by qRT-PCR. In brief, total RNA was extracted from T cells using TRIzol reagent (Invitrogen). The cDNA was synthesized using the RevertAid H Minus First Strand cDNA Synthesis Kit (Thermo Fisher Scientific, Waltham, MA, USA). Afterwards, cDNA was used to perform qRT-PCR analysis with the DyNAmo Flash SYBR Green qPCR Kit (Thermo Fisher Scientific). The 2^−ΔΔCt^ method was utilized to calculate the relative expression. GAPDH was used as the internal control.

### 2.5. Western Blotting

Antibodies used for Western blotting including the primary antibodies (JAK3, pSTAT5, STAT5, and GAPDH) and the HRP-conjugated secondary antibody were all procured from Abcam (Cambridge, UK). The procedures were performed as follows: proteins from T cells were initially lysed with RIPA buffer. We then made detection for protein concentrations using a BCA Protein Assay Kit (Thermo Fisher Scientific). Afterwards, 10% SDS-PAGE was used to separate the proteins, followed by transfer into PVDF membranes, in which incubation is with the relevant primary antibodies (1 : 1,000) at 4°C for overnight and then the secondary antibody (1 : 5,000) for 1 h at room temperature. GAPDH was used as the internal control. Immunoblottings were visualized using an ECL detection kit (Amersham Biosciences, Sweden).

### 2.6. Treatments for Patients

After isolation of T cells, all the patients were further divided into the morphine group (1 mg/kg/day; i.v.) and the Mor + Ket group (morphine: 1 mg/kg/day; ketamine: 1 mg/kg/day; i.v.) in accordance with the principles of the randomized and double-blind method. Each group contains 10 CC patients. Pain levels were assessed using a patient self-administered numeric pain intensity scale (NPIS) at introduction time (T0) and 2 h (T1), 24 h (T2), and 48 h (T3) after treatment initiation. The scale ranged from 0 (no pain) to 10 (worst pain possible) [22]. The percentages of CD4^+^ and CD8^+^ in blood samples (3 mL) were calculated using a flow cytometer (BD Biosciences) (Table 1).

### 2.7. Statistical Analysis

Data in this study were shown as mean ± SD. SPSS 23.0 software was used to perform statistical analyses. Student's *t*-test, one-way ANOVA followed by Tukey's multiple comparisons test, and two-way ANOVA followed by Tukey's multiple comparisons test or Sidak's multiple comparisons test were used to analyse the experimental data in this study. Significance difference was considered when *P* < 0.05.

## 3. Results

### 3.1. Morphine in Combination with Ketamine Relieves Pain but Suppresses Immune Function for CC Patients

**Figure 1:**
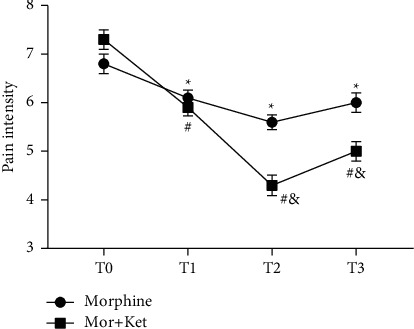
Changes in pain scores over the time between the two groups. T0, introduction time; T1, 2 h; T2, 24 h; T3, 48 h, after treatment initiation. ^*∗*^*P* < 0.05, ^#^*P* < 0.05 vs. the T0 period. ^&^*P* < 0.05 vs. the Mor (T2-T3 period) group.

We initially investigated the effects of morphine alone or in combination with ketamine on cancer pain for CC patients. As shown in Figure 1, we found that both morphine alone and morphine in combination with ketamine could significantly relieve cancer pain compared to pretreatment (*P* < 0.05). Interestingly, CC patients in the Mor + Ket group had a relatively lower pain score compared with that in the Mor group from T2 to T3 (*P* < 0.05). The immune function in patients of these two groups was further assessed. We found that for patients received combination treatments, CD4^+^ percentage and CD4^+^/CD8^+^ ratio at the postintervention period were remarkably decreased relative to those of preintervention (*P* < 0.05, Table 1). Meanwhile, similar patterns were observed in the patients who received only morphine treatment (*P* < 0.05).

### 3.2. Morphine Combined with Ketamine Decreases CD4^+^/CD8^+^ Ratio In Vitro

**Figure 2:**
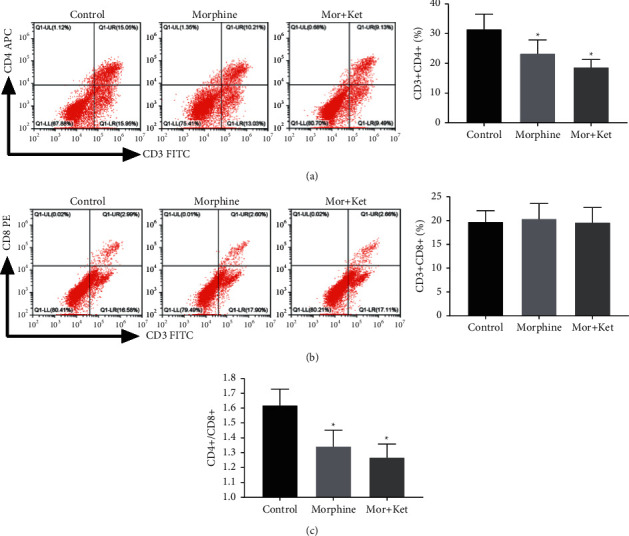
Morphine combined with ketamine decreases CD4^+^/CD8^+^ ratio in vitro. (a) The percentage of CD4^+^ assessed by flow cytometry analysis. (b) The percentage of CD8^+^ assessed by flow cytometry analysis. (c) Analysis for CD4^+^/CD8^+^ ratio. ^*∗*^*P* < 0.05 vs. the control group.

The effects of combination treatments on CD4^+^, CD8^+^, and CD4^+^/CD8^+^ ratio were further validated in vitro. The results of flow cytometry analysis demonstrated that CD4^+^ percentages and CD4^+^/CD8^+^ ratio in both morphine and Mor + Ket groups were reduced by contrast to the control group (*P* < 0.05, Figures 2(a) and 2(c)); more importantly, there were no significant differences between the morphine and Mor + Ket groups in CD4^+^ percentages and CD4^+^/CD8^+^ ratio. Meanwhile, CD4^+^/CD8^+^ ratio in the morphine, Mor + Ket, and controls groups also exhibited no significant differences (Figure 2(b)).

### 3.3. Morphine and Ketamine Inhibit the Secretion of IL-2, IFN-*γ*, and IL-17 In Vitro

**Figure 3:**
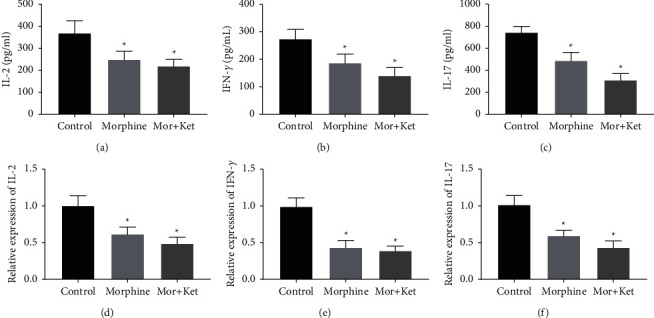
Morphine and ketamine inhibit the secretion of IL-2, IFN-*γ*, and IL-17 in vitro. (a) The level of IL-2 measured by ELISA. (b) The level of IFN-*γ* measured by ELISA. (c) The level of IL-17 measured by ELISA. (d) The mRNA expression of IL-2 detected by qRT-PCR. (e) The mRNA expression of IFN-*γ* detected by qRT-PCR. (f) The mRNA expression of IL-17 detected by qRT-PCR. ^*∗*^*P* < 0.05 vs. the control group.

It is well known that IL-2, IFN-*γ*, and IL-17 were crucial cytokines for T cell function [23–25]. Therefore, we further determined the expression levels of IL-2, IFN-*γ*, and IL-17 in the above three groups. As shown in Figures 3(a)–3(f), both the results of ELISA and qRT-PCR indicated that the levels of IL-2, IFN-*γ*, and IL-17 in the morphine group and Mor + Ket group were all suppressed compared to those of control groups (*P* < 0.05); at the same time, no significant differences were found between the morphine group and Mor + Ket group.

### 3.4. Opioid Antagonist Naltrexone Eliminates the Inhibitory Effects of Morphine and Ketamine on IL-2, IFN-*γ*, and IL-17 In Vitro

**Figure 4:**
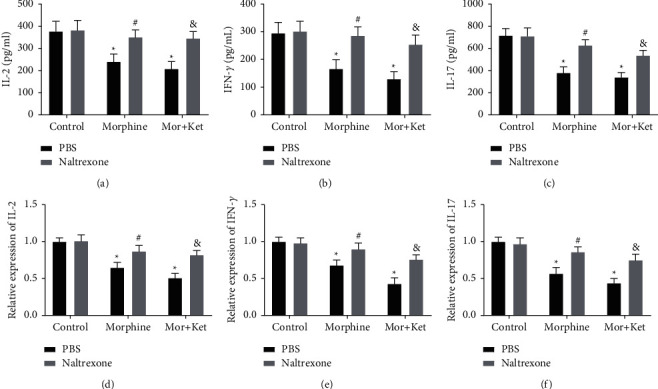
Opioid antagonist naltrexone eliminates the inhibitory effects of morphine and ketamine on IL-2, IFN-*γ*, and IL-17 in vitro. (a) The level of IL-2 after naltrexone treatment measured by ELISA. (b) The level of IFN-*γ* after naltrexone treatment measured by ELISA. (c) The level of IL-17 after naltrexone treatment measured by ELISA. (d) The mRNA expression of IL-2 after naltrexone treatment detected by qRT-PCR. (e) The mRNA expression of IFN-*γ* after naltrexone treatment detected by qRT-PCR. (f) The mRNA expression of IL-17 after naltrexone treatment detected by qRT-PCR. ^*∗*^*P* < 0.05 vs. the control (PBS) group. ^#^*P* < 0.05 vs. the morphine (PBS) group. ^&^*P* < 0.05 vs. the Mor + Ket (PBS) group.

Opioid antagonist naltrexone can specifically inhibit the effects of opioids such as morphine [26]. Therefore, naltrexone (10^−8^ M) was used to treat T cells. We found that naltrexone alone had a little effect on the expression levels of IL-2, IFN-*γ*, and IL-17 (Figures 4(a)–4(f)). As expected, naltrexone treatment eliminates the inhibitory effects of morphine alone or morphine in combination with ketamine on the release of IL-2, IFN-*γ*, and IL-17 (*P* < 0.05). Interestingly, the alleviative effects caused by naltrexone in the morphine group and Mor + Ket group exhibited no significant differences.

### 3.5. Naltrexone Reverses the Suppressive Effects of Morphine and Ketamine on JAK3/STAT5 Pathway In Vitro

**Figure 5:**
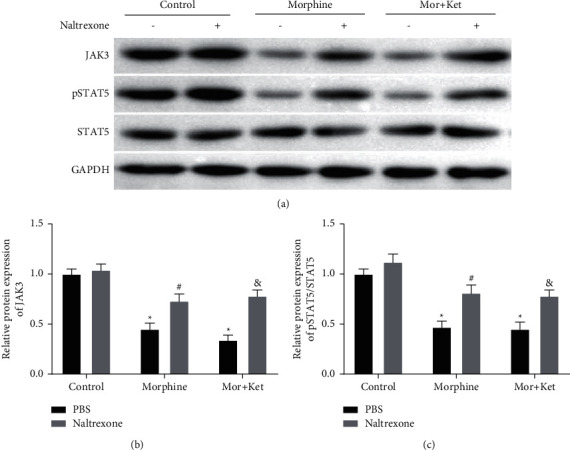
Naltrexone reverses the suppressive effects of morphine and ketamine on the JAK3/STAT5 pathway in vitro. (a) The Western blot assay images for the levels of JAK3, pSTAT5, and STAT5 in T cells. (b) The protein level of JAK3 in T cells measured by Western blot assay. (c) The protein level of pSTAT5/STAT5 in T cells measured by Western blot assay. ^*∗*^*P* < 0.05 vs. the control (PBS) group. ^#^*P* < 0.05 vs. the morphine (PBS) group. ^&^*P* < 0.05 vs. the Mor + Ket (PBS) group.

TheJAK3/STAT5 pathway is known to be associated with opioids and T cell function [27–29]. Therefore, the interaction between combination treatments and this pathway was investigated. As shown in Figures 5(a)–5(c), the protein levels of JAK3 and pSTAT5/STAT5 were suppressed not only in the morphine group but also in the Mor + Ket group (*P* < 0.05). Unsurprisingly, addition of naltrexone reversed the suppressive effects caused by morphine or Mor + Ket on JAK3 and pSTAT5/STAT5 (*P* < 0.05), and there were also no significant differences between the morphine and Mor + Ket groups.

### 3.6. Morphine and Ketamine Restrain the Levels of IL-2, IFN-*γ*, and IL-17 via JAK3/STAT5 Pathway In Vitro

**Figure 6:**
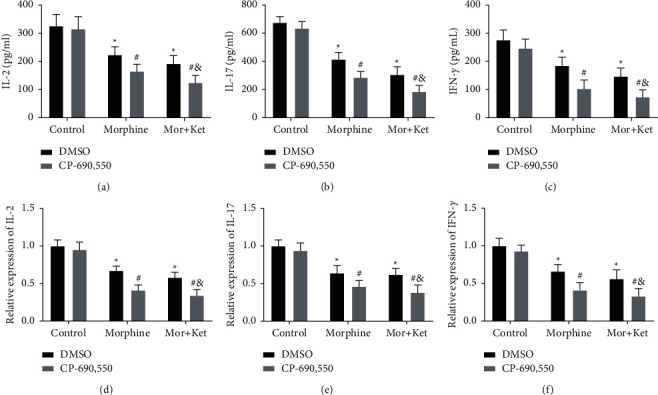
Morphine and ketamine restrain the levels of IL-2, IFN-*γ*, and IL-17 via the JAK3/STAT5 pathway in vitro. (a) The level of IL-2 after CP-690,550 treatment measured by ELISA. (b) The level of IL-17 after CP-690,550 treatment measured by ELISA. (c) The level of IFN-*γ* after CP-690,550 treatment measured by ELISA. (d) The mRNA expression of IL-2 after CP-690,550 treatment detected by qRT-PCR. (e) The mRNA expression of IL-17 after CP-690,550 treatment detected by qRT-PCR. (f) The mRNA expression of IFN-*γ* after CP-690,550 treatment detected by qRT-PCR. ^*∗*^*P* < 0.05 vs. the control (DMSO) group. ^#^*P* < 0.05 vs. the control (CP-690,550) group. ^&^*P* < 0.05 vs. the Mor + Ket (DMSO) group.

CP-690,550 (500 nM), an inhibitor of JAK3 [30], was added to T cells to determine the relationship between morphine combined ketamine and JAK3/STAT5 pathway in T cell function cytokines. As shown in Figures 6(a)–6(f), the levels of IL-2, IL-17, and IFN-*γ* in morphine and Mor + Ket groups were remarkably declined by CP-690,550 treatment (*P* < 0.05). In addition, there were also significant differences between the Mor + Ket + DMSO group and Mor + Ket + CP-690,550 group in the levels of IL-2, IL-17, and IFN-*γ* (*P* < 0.05).

## 4. Discussion

As a kind of opioid analgesic, morphine is generally used for the treatment of cancer pain [10–12]. But it is regrettable that the application of morphine either in laboratory experiments or in clinical practice leads to some side effects called opioid-induced hyperalgesia, which enhance the tolerance to opioids and suppress immune functions [13–18]. Some adjuvants in combination with morphine are considered to attenuate pain to a greater extent, reduce morphine dosage, and decrease the occurrence rate of adverse effects caused by morphine treatment alone [31, 32]. Numerous studies have reported that ketamine may have some synergistic effects with morphine and relieve the morphine-induced detrimental effects [19–21, 33]. In this study, we investigated the interaction between drug combination and the JAK3/STAT5 pathway in immune functions, and our findings revealed that morphine and ketamine may attenuate cancer pain and suppress immune functions through regulation of the JAK3/STAT5 pathway.

In this study, we initially assessed the pain intensity of CC patients after injection of morphine alone or morphine-ketamine combination. Pain intensity in the morphine group was distinctly reduced at 2 h. However, a recent study conducted by Matsuoka et al. reported that the time point that relieves pain is on the 8^th^ day [12]. We believed that different routes of administration may affect the time point and duration of pain relief. Compared to the morphine group, pain intensity in the Mor + Ket group was significantly decreased 24 h after treatment. Similarly, Salas et al. also find that ketamine and morphine by continuous intravenous infusion for 1 day can decline the score of NPIS [34]. Based on these results, we speculated morphine in combination with ketamine is more effective to improve cancer pain compared to morphine alone. In addition, decreased CD4^+^ percentage and CD4^+^/CD8^+^ ratio in CC patients of these two groups were also observed after intervention. Therefore, further in vitro experiments were performed. Through flow cytometry analysis, as expected, we found the percentage of CD8^+^ was relatively stable in both morphine and Mor + Ket groups. However, CD4^+^ percentage and CD4^+^/CD8^+^ ratio in these two groups were reduced compared to the controls, but with no significant differences between the two treatments, which indicated that although morphine-ketamine combination can attenuate cancer pain more effectively, the inhibitory effects on the levels of T cells are not enhanced. Some cytokines such as IL-2, IFN-*γ*, and IL-17 are important for T cell function [23–25]. The levels of IL-2, IFN-*γ*, and IL-17 were further determined. As shown in Figure 3, the levels of these cytokines and the corresponding mRNA expression were all inhibited in both morphine and Mor + Ket groups, which is consistent with the previous study [20]. Similar to the results of T cell, no significant differences were found in these two groups. In addition, we demonstrated that opioid antagonist naltrexone reversed the inhibiting effects of morphine or morphine-ketamine on the levels of IL-2, IFN-*γ*, and IL-17. All these data suggested that both morphine and morphine-ketamine combination indeed affect the secretion of IL-2, IFN-*γ*, and IL-17 and thus regulates the expression of T cells.

The JAK3/STAT5 pathway contains two main protein families, including JAKs and STATs [27]. Among these proteins, JAK3 is specifically located in T cells and shares the same receptor with IL-2 [35]. Besides, JAK3 promotes the phosphorylation of STAT5 to modulate gene expression [36]. In terms of STAT5, increasing studies have indicated the associations between STAT5 and IFN-*γ*/IL-17 [37–39]. In the current study, we found that both morphine and morphine-ketamine suppressed the protein levels of JAK3 and pSTAT5/STAT5 and naltrexone reversed these situations. We speculated that morphine in combination with ketamine may directly interact with the JAK3/STAT5 pathway to regulate immune functions. Our data report that CP-690,550, a JAK3 inhibitor, further enhanced the suppressive effects of morphine-ketamine combination on the levels of IL-2, IFN-*γ*, and IL-17 and further validated this assumption. Therefore, we believed that compared to morphine alone, morphine and ketamine can relieve cancer pain of CC patients more effectively via the JAK3/STAT5 pathway without reducing immune function additionally.

In a word, morphine in combination with ketamine improves cancer pain and suppresses immune function via the JAK3/STAT5 pathway. These findings clarify the action mechanism of drug combinations on the treatments of cancer pain in CC patients. Support for the use of morphine-ketamine combination may be strengthened by understanding the action mechanism and establishing clinical indications and appropriate recommendations.

## Figures and Tables

**Table 1:** Percentages of CD4^+^ and CD8^+^ and CD4^+^/CD8^+^ ratio of the patients in the two groups.

	Morphine (*n* = 10)		Mor + Ket (*n* = 10)	
	Preintervention	Postintervention	Preintervention	Postintervention
CD4^+^(%)	27.21 ± 5.54	21.32 ± 4.25^*∗*^	26.84 ± 5.34	18.75 ± 6.48^#^
CD8^+^(%)	18.57 ± 4.35	18.43 ± 5.94	19.86 ± 3.17	20.35 ± 6.04
CD4^+^/CD8^+^	1.37 ± 0.32	1.02 ± 0.25^*∗*^	1.35 ± 0.38	0.87 ± 0.43^#^
Age (years)	56.32 ± 8.25		57.16 ± 6.73	
BMI (kg/m^2^)	21.36 ± 3.52		21.77 ± 3.04	

BMI, body mass index. ^*∗*^*P* < 0.05, ^#^*P* < 0.05 vs. the corresponding preintervention group.

## Data Availability

The datasets used and/or analyzed during the current study are available from the corresponding author upon request.
